# The Role of Velocity Based Training in the Strength Periodization for Modern Athletes

**DOI:** 10.3390/jfmk3040055

**Published:** 2018-11-16

**Authors:** Aristide Guerriero, Carlo Varalda, Maria Francesca Piacentini

**Affiliations:** 1 Department of Human Movement and Sport Sciences, University of Rome “Foro Italico”, 00135 Rome, Italy; 2 Italian Weight Lifting Federation, 00196 Rome, Italy; 3 Brasil Rugby, Sao Paulo 01407-200, Brazil

**Keywords:** strength, velocity, linear transducer

## Abstract

Resistance training (RT) is considered the most important method to improve the athlete’s strength and rate of force development (RFD). In the last decade, the importance of monitoring velocity during RT has drastically grown, because of an increased availability of linear position transducers (LPT) and inertial measurement units (IMU). The purpose of this review is to analyze the existing literature on testing techniques and performance strategies used to enhance strength and power performance of elite athletes, by monitoring the velocity of resistance training. The authors focus in particular on the level of effort of resistance training defined by velocity; how the loss of velocity correlates with the degree of fatigue and how it can be used to enhance the performance of competitive athletes; the use of LPT as part of the daily routine of the strength and conditioning programs in competitive sport. It is therefore critical for the sports scientists to have a correct understanding of the basic concepts of the velocity-based training and their application to elite sports. The ultimate goal is to give some indications on the velocity-based resistance training integration in the programs of different sports in the high performance environment.

## 1. Introduction

Resistance training (RT) is an effective method to induce changes in muscular strength, hypertrophy, and power [1]. In order to optimize RT programs, specifically designed to increase the athletic performance of different sports disciplines, coaches can manipulate many variables such as load, the number of sets and repetitions, types of exercise, order and velocity of movement which all induce different physiological and neuromuscular adaptations [2]. Normally training intensity and volume have been the most studied topics while movement velocity has been often overlooked [3]. However, enhancing the rate of force development (RFD), a measure of how fast an individual can develop force is essential in most Olympic sports. RFD has been shown to increase after explosive strength training and is, therefore, used as a measure of the effectiveness of a training program (for review see [4]).

Until recently exercise intensity and degree of effort have been prescribed as the percentage of the one repetition maximum (1RM) in different exercises, allowing the estimation of the relative load of different numbers of repetitions and sets [1]. An alternative method gaining more attention especially when the improvement of sport related performance is the main goal is normalizing intensity based on the measure of velocity during the concentric portion of the repetition cycle of the major strength exercises together with the creation of the velocity/load profile.

However, this method is still debated [5]. Differences in the utilization of one method over the other depend on the number and level of the athletes, and training environment. Prescribing intensity on the basis of percentage of the one repetition maximum (% 1RM) allows an optimal training individualization and at the same time identifies the athlete’s progression. For novice athletes, the use of the percentage of 1RM is easier to integrate and to apply [6], however, the daily oscillation of the 1RM value and the necessity to test 1RM for all the different movements included in the training program, poses some disadvantages. Moreover, 1RM measurement is time consuming and impractical for large groups (as teams) [7], also if novel and safer methodologies for indirect assessment have been proposed [8]. Direct assessment of 1RM may be associated with injury if performed incorrectly or by novice subjects,

Another indicator commonly used to identify the intensity of resistance training is the maximum number of consecutive repetitions of one specific exercise with specific load (nRM) performed by an individual [9,10]. Assuming that the subject performs the maximum number of concentric movements, it is possible to determine the relative intensity of the effort and the number of repetitions that can be performed. This method eliminates the necessity to continuously monitor 1RM, however it implies that athletes train with repetitions to failure, a method that has been shown to be in some cases counterproductive by inducing excessive fatigue and possibly interfering with the adaptation process [10].

More recently, the movement velocity of repetitions has been extensively studied as a way of monitoring exercise intensity [10]. This method, known as velocity based training (VBT) allows to estimate the % 1RM from the actual velocity of each repetition, without performing demanding maximal tests to adjust training loads. This method allows estimating the daily readiness (or daily 1RM) and monitors the decrease in velocity within each set to manage the accumulation of fatigue [1].

Previous studies with strength and top-level athletes have related the velocity of the bar and load lifted, proving the validity of the estimation of 1RM percentage based on movement velocity [1,11]. The two necessary key components to prescribe VBT are the percentage of loss of velocity and the mean concentric velocity of the fastest repetition (which is related to the loading magnitude) [1]. These variables are exercise specific and can lay down the foundation of precise individualization of training intensity. In fact, as identified in previous studies, the movement velocity is inversely proportional to the load lifted while it is directly proportional to the effort, meaning that the concentric velocity of the lifting will drop when the load is closer to 1RM and during the execution of sets with multiple repetitions due to fatigue [7].

Movement velocity is exercise dependent and it is often prescribed as the mean velocity (MV) or as mean propulsive velocity (MPV) of the concentric phase, the velocity achieved during the execution of the exercise is the result of the force applied [6]. Interestingly, MPV attained with each % 1RM is a stable indicator of exercise intensity despite improvements in 1RM [1].

Most studies analyzing resistance training velocity have also found that an unintentional decrease in force and velocity applied is observed along the completion of given sets [9]. In particular the loss of velocity between the repetition and the sets of the same exercises could be used as an indicator to monitor fatigue levels [12].

The most common accurate device used to measure velocity during resistance training is the linear position transducer (LPT). This kinematic device normally has the form of a processing unit with a retractable cable, and directly measures the vertical displacement of the cable that is attached to the barbell, dumbbell, gym equipment or the athletes themselves. Normally LPT is connected to a display in order to have live feedback on velocity [13]. The potential limitations of including loss velocity monitoring with LPT in the daily training routine are the expense of more accurate devices, lower control of the training process and a more complex tracking method for coaches to manage.

It is still not clear which method between percentage based and movement velocity based is more appropriate and if fast concentric and eccentric movements determine greater cross-sectional areas of the muscle compared with the normal tempo or slow movement strength training [5].

The prescription of resistance training based on the movement velocity is widely used by recreational populations [5], but elite athletes in high performance environment integrate the aforementioned method in different ways.

Therefore, the purpose of the current paper is to review the existing literature on the effects of VBT and the common scheme methods utilized by elite athletes. The information extrapolated from this review may help define scientific guidelines for strength coaches, athletic trainers and technical coaches.

## 2. Materials and Methods 

### 2.1. Experimental Approach to the Problem

**Figure 1:**
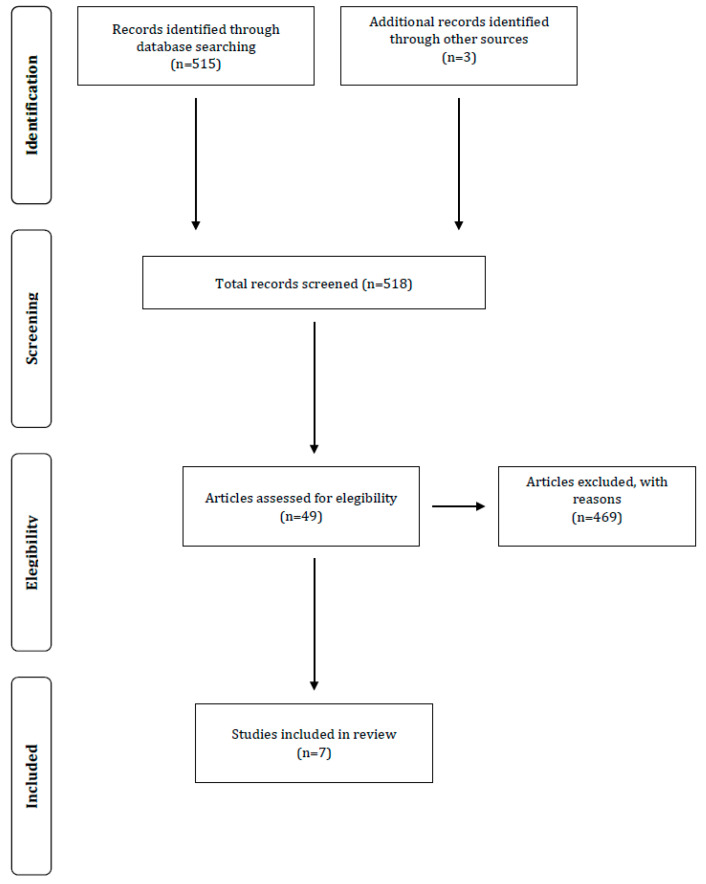
Flow diagram of search process.

The literature search of English-language journals was conducted on electronic databases up to July 2018 (Figure 1). The search included: Pub Med, SPORTDiscus and Medline. The following keywords were used to carry out the search: strength training AND velocity loss, resistance training AND velocity loss, linear position transducer. Search terms were modified accordingly to fit the requirements of the database used.

### 2.2. Screening Process and Inclusion Criteria 

The screening process was conducted using the following method: (1) all articles obtained were selected by title and duplicates were deleted; (2) an integral reading of the remaining studies was conducted, and those that were deemed outside the scope of the current review were excluded.

Criteria for inclusion were: (a) studies published in English; (b) full texts available; (c) studies involving only competitive athletes; (d) studies including a resistance training protocol of more than four weeks; (e) studies that included any resistance training utilizing different loads of the 1RM, during which velocity was monitored, and (f) studies that utilized a LPT to monitor velocity.

Studies including IMU were excluded.

The studies that included untrained subjects or individuals with musculoskeletal injuries were excluded.

## 3. Results

The database search yielded 515 potential studies (Figure 1). Only seven studies met the eligibility criteria and were included in the review (Table 1).

A total of 203 male participants aged between 16 and 35 years were part of the studies. All seven studies included competitive male athletes in three different sports: kayaking (*n* = 31), rowing (*n* = 27), and soccer/football (*n* = 28, 30, 23, 29, 25). The length of the studies went from 6 weeks to 26 weeks in total with a training frequency of 2–3 sessions per week. Training specifics of the studies are presented in Table 1.

Of the seven studies, five monitored the velocity of the barbell during squat and squat jump [3,7,9,16,17] and two studies focused on barbell velocity during bench press and bench pull [15,16]. In all studies, the subjects were instructed to complete the repetitions with the maximum intended concentric velocity. Two of the studies considered in the review [9,14], declared the velocity loss targeted during VBT (between 10% and 30%). All studies reported the presence of a high-qualified supervisor during the training session or protocols used. Three studies specified the periodization used during the intervention period: linear periodization [11,14] and traditional strength-power periodization [18]. All studies indicated concurrent training methods with more than one aspect trained at the same time: technical/tactical, power, strength, speed, and energy system development. All studies used the 1RM Test, estimated 1RM Test or Isoinertial Progressive Load Test as pre- and post-test to measure the dynamic strength index. Three studies [3,9,16] measured aerobic fitness, speed and power as part of the testing battery (Table 2). To train maximum power, barbell movement velocity of ≥0.95–1 m·s^−1^ (30% and 58% of 1RM) was used.

The main results of the studies are reported in Table 2.

The two studies that evaluated the effects of different resistance training approaches in endurance sports (kayakers and rowers) [14,15] showed both an increase in aerobic fitness, 1RM and maximal power as described in Table 2. In one study [14] training to repetition failure identified as “the inability to complete a repetition in its full range of motion” was avoided while in the study by Izquierdo-Gabarren et al., 2010 [15], training to failure or not to failure were compared. Specifically training not to failure showed larger gains in 1RM bench pull strength, muscle power output, and rowing performance [15].

Three studies [7,9,17] evaluated the effects of VBT on soccer specific performance in professional players. Training with optimum power load (OPL-1.0 m·s^−1^) showed a superior improvement in the sprint tests and similar improvements in 1RM squat, squat jump, CMJ [7]. Training according to velocity loss during each set: 15% (VL15) and 30% (VL30) showed similar improvements between the groups (VL15–VL30) in all parameters (strength, speed power and endurance capacity), while VL15 showed greater gains in CMJ [9]. Moreover, combining different light-load maximal lifting velocity weight training and plyometrics proved to be effective for improving 1RM, CMJ, and sprint performance [17]. The last two studies taken in consideration [3,16] evaluated the effects of VBT on soccer specific performance of elite young soccer players (mainly U16–U19). Both studies included a RT of 16 and 26 weeks length respectively, with moderate loads (50–65% 1RM) concomitant to the soccer training. Players that included RT showed significant improvements in strength, power and maximal aerobic speed.

## 4. Discussion

The present review analyzed the results of seven studies that evaluated the effects of the integration of VBT in the training program of elite athletes of different sports disciplines using the LPT. The LPT and IMU are both commonly used to monitor movement velocity; modern IMUs can be worn around the wrist or attached directly to the barbell. The commercial devices include an integrated gyroscope and accelerometer, and they estimate the barbell velocity through specific algorithms for each exercise [19] while LPT measure directly the vertical displacement of a cable (attached to the barbell) [13].

Although IMU can be inexpensive compared to LPT, they have been shown to record lower velocities compared to three-dimensional motion capture software that is considered the gold standard measurement. The LPT showed almost a perfect correlation with three-dimensional motion capture software for mean concentric velocity (*R*^2^ = 0.985) [19]. For this reason, we included only studies that utilized LPT as a measuring device.

Based on the results from this review it appears that resistance training periodization (≥4 weeks) and velocity monitoring can be very effective in enhancing sport specific performance together with endurance and power training in competitive athletes during the in-season period. The basic concept of the velocity-based training is the assumption that the subjects or the athletes always complete the repetitions with maximal voluntary velocity. Considering the time constraints of elite athletes and the necessity to train different aspects of sport specific performance, it is important to avoid the so called “interference phenomenon” during concurrent training and avoid nonfunctional overreaching.

Furthermore, if we consider the velocity-based training integrated into a periodization model, a wide range of movement velocities can be prescribed to maximize athletic performance. The velocity of the concentric phase is exercise specific [2]. According to the current review, the most common velocities used to train different points of the force-velocity relationship are 45% 1RM (1.20 m·s^−1^) up to 90% 1RM (0.40 m·s^−1^). Moreover, muscle power ability is one of the most determinant aspects of different sports performances [18], it is, therefore, important to identify the load that elicits the velocity of 1.00 m·s^−1^; this load has been shown to optimize mean propulsive power independently of the exercise [20,21].

In particular, power output is often a key aspect during the competitive period of most of individual and team sports. This concept is well addressed by the study of Loturco et al. [7] where both methods of resistance training (load eliciting 1.00 m·s^−1^ vs. traditional wave loading) are compared. While the traditional strength training periodization was originally based on the 1RM assessment and relative percentage, the OPL uses the bar velocities to determine the optimum range of loads to maximize power output. The group that completed the strength/power periodization completed a classic wave loading progress from 60% to 90% of 1RM, comprehensive of an accumulation, transformation and realization phase. During this process, the physiological adaptations from the first two phases would be transferred to muscular power due to the manipulation of volume and intensity. The second group performed squat jumps with OPL. Although the obtained results show similar improvements in 1RM, squat and change of direction speed, the group that trained with high velocity showed more marked speed improvements despite spending less time in the weight room. These speed improvements on the longer distances (10 m and 20 m) may be affected by neuromechanical and biomechanical factors and most importantly, it seems that the OPL group was capable of developing both strength and power, by optimizing the training intervention.

Another effective way of enhancing speed, power and strength are by combining exercises performed at maximal velocity (≥1.00 m·s^−1^) with plyometric exercises [17]. This combination seems to transfer into superior improvements on sport-related markers like sprint ability on the 20 m when comparing it with resistance training alone (−2.93% improvement versus −1.50%) [17].

One of the key aspects to consider when implementing the velocity monitoring in the daily training is the velocity loss targeted in the set and between the sets compared to the first repetition (usually the fastest) [9]. A previous study [12] has shown that along the completion of multiple repetitions an unintentional decrease in velocity, power, and force is observed. Monitoring velocity loss can be a different method to estimate the level of mechanical and physiological fatigue. The study by Pareja-Blanco et al. [9] compared the effects of 2 RT programs by using the velocity loss during the set as independent variable (15% or 30%). The authors reported similar gains in squat performance with the first group experiencing greater gains in CMJ. The common factor is again that the group that trained with a loss of velocity lower than 15%, spent less time in the weight room, performed a minor number of repetitions and probably experienced a lower level of fatigue. Other studies reported that for upper body exercises the loss of velocity should not exceed 10% [14] in order to attain very high power outputs for a few selected repetitions. The results of these studies indicate that monitoring carefully both MPV and loss of velocity during the sets, avoid performing unnecessary slow and fatiguing repetitions that may tamper training effects or cause excessive fatigue and optimizes maximal power in already highly trained athletes.

One of the most common ways to determine exercise intensity and load until now has been to determine the maximum number of repetitions that can be performed with a given submaximal weight [1]. Training to failure has been shown to not necessarily improve the magnitude of strength gains (specifically in the elite) and may cause excessive strain for subsequent training sessions. In fact, recent studies [9,15] showed that training not to failure with a moderate volume of repetitions could elicit greater enhancements in strength, muscle power and also rowing performance compared to training to failure in highly trained athletes. The authors reported that the group that trained with a reduced training volume presented similar sport performance gains with half of the work. In particular, to improve the performance in sports with a high demand of aerobic endurance and muscle strength, optimizing the number of repetitions per set by VBT can be the effective option for high-level athletes specifically when performing concurrent training [15]. These results suggest that moderate-volume high-intensity stimuli are needed to induce further power gains with experienced highly trained athletes when the concurrent development of both strength and endurance are important [16].

In two of the studies [14,15] the authors used the linear periodization with the integration of the velocity-based training for the bench press and prone bench pull. One study of 8 weeks length implemented a linear periodization concurrent strength and endurance training for rowers, while the second study was 12 weeks periodized program combining strength and endurance training with kayakers. These two studies reflect what can be some real challenges of a high-performance program where the interference between endurance training and resistance training is unavoidable. In the study by Garcia-Pallares et al. [14] despite the training time spent to improve endurance was triple the resistance training time, both strength and power markers of bench press and prone bench pull improved consistently (4.2% and 14.4%). In particular, this was obtained with a careful periodized training where mixing possible interfering objectives was avoided. During the first period of training the main objective was the improvement of muscle hypertrophy, while for the successive period more sessions were include focusing on the energy system development. In the last training period the concurrent training of strength/power and aerobic power was attainable due to the strength stimulus being purely neural by monitoring resistance training velocity. The periodized training program used in the study avoided mixing the two objectives of muscle hypertrophy and maximal aerobic power because those models of training lead to opposing physiological adaptations. VBT in fact stresses the neural system without high metabolic demands [14]. Four of the studies [3,7,14,16] considered in this review planned the RT sessions before the technical/tactical and aerobic endurance sessions highlighting the importance of the timing to maximize the RT sessions.

There are several limitations that need to be taken into account when interpreting the results of these seven studies. Firstly, the small number of studies that met the inclusion criteria and a small amount of data analyzed. Manipulating velocity and its effects are hard to quantify due to the concurrent training of different aspects (plyometrics, speed, agility, and fitness). Secondly, the subjects of the studies varied in terms of age, sports and level of resistance training experience, which could create a different level of adaptation and not all studies had a CG. Future research studies comparing velocity loss or resistance training with velocity monitoring, 1RM percentage training method and nRM method or repetition to failure, with competitive athletes are needed. More studies monitoring movement velocities of different multi joints exercises with elite athletes and female athletes are lacking.

## 5. Practical Applications

The findings of this review can provide some guidance regarding the implementation of velocity-based training in an elite training environment:

- Resistance training with loads that elicit the velocity of the movement of ≈1.00 m·s^−1^ are the most appropriate loads to improve mean propulsive power.
- Resistance training for lower body exercises with a loss of movement velocity in the set of 15%–10% of the fastest repetitions (normally the first of the set) is recommended, while 5%–10% is sufficient for upper body exercises.
- Combining resistance training with maximal movement velocity (≥1.00 m·s^−1^) and plyometric exercises is an effective method to better transfer the strength gains into sport performance (in particular jumping and acceleration).
- The use of low repetitions with low load has no interference with aerobic endurance and induces specific explosive strength training adaptations. However, RT needs to be performed prior to the endurance training sessions according to the training final goals.

## Figures and Tables

**Table 1:** Overview of studies meeting inclusion criteria.

Study	Participants	Training Comparison Groups	Set × Repetition	Resistance Training Exercise (s)	Duration of Intervention; Weekly Training Frequency	Periodization Model	Equipment
Garcia-Pallares et al. (2009) [14]	Kayakers world class men (*n* = 11)	K: resistance training, fitness sport specific;	4–5 × 8–10; 3–4 × 3–4; 4–5 × 5–8	Bench Press and Prone Bench Pull	12 weeks; 3×	Linear Periodization	LPT
Gonzalez-Badillo et al. (2015) [3]	Young elite soccer players men (*n* = 44)	U16-U18 (EXP): resistance training, power, speed/agility technical/tactical; U21 (CG): technical/tactical training;	2 × 8; 3 × 8; 3 × 6; 2 × 6; 3 × 4; 4 × 6	Squat	26 weeks; 2×	Not specified	LPT
Izquierdo-Gabarren et al. (2010) [15]	Rowers men (*n* = 43)	4RF- 4RNF-2RNF (EXP): resistance training, fitness sport specific; CG: none;	3 × 5; 4 × 5; 3 × 4; 4 × 4; 3 × 3; 4 × 3; 3 × 2; 4 × 2;	Prone Bench Pull	8 weeks; 2×	Linear Periodization	LPT
Lopez-Segovia et al. (2010) [16]	U19 soccer players men (*n* = 37)	Team A (EXP): resistance training, power, technical/tactical, speed; Team B (CG): technical/tactical, power;	8 × 4; 6 × 6; 3 × 6; 4 × 4; 2 × 4; 3 × 4; 4 × 4; 2 × 4; 7 × 4; 5 × 4;	Squat	16 weeks; 2×	Not specified	LPT
Loturco et al. (2016) [7]	Professional soccer players men (*n* = 23)	TSP (CG): resistance training, power, technical/tactical; OPL (EXP): resistance training, power, technical/tactical;	6 × 10; 6 × 8; 6 × 6; 6 × 4	Squat and Squat Jump	6 weeks; 3×	Traditional Periodization/Optimum Load	LPT
Pareja-Blanco et al. (2017) [9]	Professional soccer players men (*n* = 20)	VL15: resistance training, training sport specific (not specified); VL30: resistance training, training sport specific (not specified);	2 × 3;	Squat	6 weeks; 3×	Not specified	LPT
Rodriguez-Rosell et al. (2017) [17]	Semiprofessional soccer players men (*n* = 30)	FSG (EXP): resistance training; COM (EXP): resistance training, power, speed; CG: none;	2 × 6; 3 × 6; 3 × 5; 2 × 5; 3 × 4; 2 × 4;	Squat	6 weeks; 3×	Not specified	LPT

K = Kayakers; U16 = Under 16; U18 = Under 18; U21 = Under 21; 4RF = Group with four exercises leading to failure; 4NRF = Group with four exercises not leading to failure; 2NRF = Group with two exercises not leading to failure; TSP = Group following strength-power periodization; OPL = Group following optimum-load periodization; VL15 = Group with velocity loss of 15%; VL30 = Group with velocity loss of 30%; FSG = Group completing resistance training alone; COM = Group completing resistance training combined with power and speed training; EXP = Experimental Group; CG = Control Group; LPT = Linear Position Transducer.

**Table 2:** Overview of the results.

Study	Exercises	Strength Test (Pre-Post)	Power Test (Pre-Post)	Speed Test (Pre-Post)	Fitness Test (Pre-Post)
Garcia-Pallares et al. (2009) [14] K	Prone Bench Pull/Bench Press	1RM BP (4.2% ⇑; *p* < 0.05) 1RM PBP (5.3% ⇑; *p* < 0.05)	V45% BP (14.4% ⇑; *p* < 0.0001) V45% PBP (10% ⇑; *p* < 0.0001)	NA	KE VO2max (9.5% ⇑; *p* < 0.05)
Gonzalez-Badillo et al. (2015) [3] U16 (EXP)	Squat	V1LOAD (41.7.4 ± 9.3 − 69.9 ± 12.5 ⇑; *p* < 0.000)	CMJ (35.4 ± 3.9 − 39.1 ± 4.9 ⇑; *p* < 0.000)	T20 (2.99 ± 0.10 − 2.97 ± 0.09 ⇑; *p* < 0.14)	MAS (15.9 ± 0.7 − 16.2 ± 0.8 ⇑; *p* < 0.02)
U18 (EXP)		V1LOAD (51.6 ± 10.7 − 66.6 ± 10.1 ⇑; *p* < 0.000)	CMJ (38.4 ± 3.0 − 41.3 ± 4.5 ⇑; *p* < 0.000)	T20 (2.96 ± 0.10 − 2.92 ± 0.10 ⇑; *p* < 0.02)	MAS (15.8 ± 1.0 − 16.0 ± 0.8 ⇑; *p* < 0.12)
U21 (CG)		V1LOAD (53.1 ± 4.9 − 65.9 ± 2.2 ⇑; *p* < 0.000)	CMJ (37.1 ± 3.7 − 38.1 ± 3.5 ⇑; *p* < 0.36)	T20 (2.97 ± 0.09 − 2.96 ± 0.10 ⇑; *p* < 0.36)	MAS (15.9 ± 0.7 – 15.9 ± 0.8 ⇔; *p* < 0.91)
Izquierdo-Gabarren et al. (2010) [15] 4RF (EXP)	Bench Press	1RM BP (2.1% ⇑)	MPO 75–85% (−3.1% and −2.7% ⇓)	NA	W4 mmol⋅L^−1^ (5.3% ⇑; *p* < 0.05) W20 min (4.6% ⇑; *p* < 0.05) W10 strokes (−0.1% ⇓; *p* < 0.05)
4NRF (EXP)		1RM BP (4.6% ⇑)	MPO 75–85% (6.6% and 6.7% ⇑)	NA	W4 mmol⋅L^−1^ (6.2% ⇑; *p* < 0.05) W20 min (7.6% ⇑; *p* < 0.05) W10 strokes (3.6% ⇑; *p* < 0.05)
2NRF (EXP)		1RM BP (0.6% ⇑)	MPO 75–85% (6.6% and 6.7% ⇑)	NA	W4 mmol⋅L^−1^ (6.8% ⇑; *p* < 0.05 W20 min (9.0% ⇑; *p* < 0.05) W10 strokes (5% ⇑; *p* < 0.05)
CG		1RM BP (−2.6% ⇓)	NA	NA	W4 mmol⋅L^−1^ (4.5% ⇑; *p* < 0.05) W20 min (4.5% ⇑; *p* < 0.05) W10 strokes (−0.8% ⇓; *p* < 0.05)
Lopez-Segovia et al. (2010) [16] Team A (EXP)	Squat	FS30 (1.27 ± 0.13 − 1.36 ± 0.12 ⇑; SE 0.72) FS50 (1.06 ± 0.12 − 3.15± 0.10 ⇑; SE 0.12) FS70 (0.78 ± 0.14 − 0.82 ± 0.16 ⇑; SE 0.27)	CMJ (35.37 ± 5.1 − 37.12 ± 4.5 ⇑; SE 0.34) CMJ20 (22.78 ± 3.6 − 24.33 ± 3.4 ⇑; SE 0.44)	T10 (1.82 ± 0.06 − 1.85 ± 0.1 ⇓; SE 0.36) T20 (3.08 ± 0.11 − 3.15 ± 0.13 ⇓; SE 0.58) T30 (4.25 ± 0.15 − 4.35 ± 0.19 ⇓; SE 0.58)	MAS (16.39 ± 0.28 − 16.291 ± 0.9 ⇑; SE 0.78)
Team B (CG)		FS30 (1.25 ± 0.1 − 1.29 ± 0.08 ⇑; SE 0.36) FS50 (0.89 ± 0.19 − 1.01 ± 0.12 ⇑; SE 0.76) FS70 (0.74 ± 0.11 – 0.87 ± 0.10 ⇑; SE 1.24)	CMJ (34.2 ± 5.1 − 35.44 ± 5.2 ⇑; SE 0.24) CMJ20 (18.93 ± 2.9 − 22.9 ± 4 ⇑; SE 1.14)	T10 (1.88 ± 0.05 − 1.85 ± 0.06 ⇑; SE-0.54) T20 (3.16 ± 0.1 − 3.15 ± 0.09 ⇑; SE-0.21) T30 (4.40 ± 0.15 − 4.36 ± 0.17 ⇑; SE-0.25)	MAS (15.72 ± 1.3 − 15.66.± 1.1 ⇑; SE-0,05)
Loturco et al. (2016) [7] TSP (CG)	Squat/Squat Jump	1RM SQ (8.1 ± 2.8% ⇑; *p* < 0.0001)	CMJ (11.4 ± 4.3% ⇑; *p* < 0.0001) SJ (13.4 ± 4.7% ⇑; *p* < 0.0001) MPP40 (3.0 ± 4.4% ⇑; *p* < 0.10)	T5 (7.2 ± 3.3% ⇑; *p* < 0.0001) T10 (3.3 ± 2.7%⇑; *p* < 0.0001) T20 (2.3 ± 2.4%⇑; *p* < 0.0001) COD (6.6 ± 1.8% ⇑; *p* < 0.0001)	NA
OPL (EXP)		1RM SQ (7.5 ± 1.9% ⇑; *p* < 0.0001)	CMJ (11.4 ± 4.3% ⇑; *p* < 0.0001) SJ (13.8.1 ± 4.2% ⇑; *p* < 0.0001) MPP40 (13.0 ± 3.5% ⇑; *p* < 0.0001)	T5 (8.0 ± 2.1% ⇑; *p* < 0.0001) T10 (7.1 ± 1.5%⇑; *p* < 0.0001) T20 (5.9 ± 0.9%⇑; *p* < 0.0001) COD (6.8 ± 2.6% ⇑; *p* < 0.0001)	NA
Pareja-Blanco et al. (2017) [9] VL15	Squat	1RM SQ (101.3 ± 18.8 − 110.3 ± 14.3 ⇑; SE 0.43) AMPV (1.19 ± 0.12 − 1.23 ± 0.09 ⇑; SE 0.35)	CMJ (33.7 ± 3.6 − 35.5 ± 5.1 ⇑; SE 0.45)	T30 (4.32 ± 0.19 − 4.30 ± 0.20 ⇑; SE 0.10)	YIRT (1390 ± 417 – 1862 ± 639 ⇑; SE 1.01)
VL30		1RM SQ (100 ± 20.3 − 106 ± 28.5 ⇑; SE 0.28) AMPV (1.16 ± 0.12 − 1.18 ± 0.13 ⇑; SE 0.16)	CMJ (34.4 ± 3.5 − 33.5 ± 3.1 ⇓; SE-0.24)	T30 (4.28 ± 0.14 – 4.27 ± 0.10 ⇑; SE 0.06)	YIRT (1611 ± 639 – 2043 ± 842 ⇑; SE 0.97)
Rodriguez-Rosell et al. (2017) [17] FSG (EXP)	Squat	1RM SQ (86.9 ± 14.2 − 101.2 ± 10.3 ⇑; 17.3%)	CMJ (37.8 ± 3.9 − 39.8 ± 4.2 ⇑; 5.15%)	T10 (1.77 ± 0.08 − 1.72 ± 0.06 ⇑; −2.67%) T20 (3.04 ± 0.11 − 2.99 ± 0.09 ⇑; −1.50%)	NA
COM (EXP)		1RM SQ (91.8 ± 14.7 − 104.4 ± 17.8 ⇑; 13.36%)	CMJ (36.3 ± 4.1 − 38.9 ± 4.7 ⇑; 7.10%)	T10 (1.78 ± 0.09 − 1.71 ± 0.08 ⇑; −3.61%) T20 (3.06 ± 0.13 − 2.97 ± 0.14 ⇑; −2.93%)	NA
CG		1RM SQ (92.5 ± 20.7 − 91.6 ± 17.9 ⇓; −0.45%)	CMJ (37.1 ± 3.8 − 37.0 ± 4.2 ⇓; −0.56)	T10 (1.77 ± 0.06 − 1.78 ± 0.06 ⇓; 0.69) T20 (3.04 ± 0.08 − 3.06 ± 0.07 ⇓; 0.67)	NA

K = Kayakers; KE VO2max = Kayak-Ergometer maximal oxygen uptake test; U16 = Under 16; U18 = Under 18; U21 = Under 21; 4RF = Group with four exercises leading to failure; 4NRF = Group with four exercises not leading to failure; 2NRF = Group with two exercises not leading to failure; TSP = Group following strength-power periodization; OPL = Group following optimum-load periodization; VL15 = Group with velocity loss of 15%; VL30 = Group with velocity loss of 30%; FSG = Group completing resistance training alone; COM = Group completing resistance training combined with power and speed training; EXP= Experimental Group; CG = Control Group; V1LOAD = Load that elicits 1 m·s^−1^; MPO = Maximal Power Output; W4 mmol⋅L^−1^ = average row power output eliciting a blood lactate concentration of 4 mmol·L^−^^1^; W20 min = average power during a 20-min all-out row test; W10strokes = power output in 10 maximal strokes; MAS = Maximal Aerobic Speed; T5 − T10 − T20 − T30 = Time 5 m − 10 m − 20 m − 30 m; FS30 − 50 − 70 = Full squat 30 kg – 50 kg – 70 kg; CMJ = Counter Movement Jump; SJ = Squat Jump; BP = Bench Press; PBP = Prone Bench Pull SQ = Squat; MPP40 = Mean Propulsive Power with 40% of Body Weight in Jump Squat; COD: Change of direction test; AMPV = Average Mean Propulsive Velocity against absolute loads common to Pre-Post tests in the squat progressive-loading test; YIRT = Yo Yo Intermittent Recovery Test Lv1; SE = Size Effect.
